# The Impact of Epstein-Barr Virus Infection on Epigenetic Regulation of Host Cell Gene Expression in Epithelial and Lymphocytic Malignancies

**DOI:** 10.3389/fonc.2021.629780

**Published:** 2021-02-25

**Authors:** Merrin Man Long Leong, Maria Li Lung

**Affiliations:** ^1^ Division of Infectious Diseases, Department of Medicine, Brigham and Women’s Hospital, Harvard Medical School, Boston, MA, United States; ^2^ Department of Microbiology, Harvard Medical School, Harvard University, Boston, MA, United States; ^3^ Department of Clinical Oncology, University of Hong Kong, Hong Kong, Hong Kong

**Keywords:** Epstein-Barr virus, epigenetics, histone modifications, DNA methylation, nasopharyngeal carcinoma, EBV-associated gastric cancer, EBV-associated lymphomas

## Abstract

Epstein-Barr virus (EBV) infection is associated with a variety of malignancies including Burkitt’s lymphoma (BL), Hodgkin’s disease, T cell lymphoma, nasopharyngeal carcinoma (NPC), and ∼10% of cases of gastric cancer (EBVaGC). Disruption of epigenetic regulation in the expression of tumor suppressor genes or oncogenes has been considered as one of the important mechanisms for carcinogenesis. Global hypermethylation is a distinct feature in NPC and EBVaGC, whereas global reduction of H3K27me3 is more prevalent in EBVaGC and EBV-transformed lymphoblastoid cells. In BL, EBV may even usurp the host factors to epigenetically regulate its own viral gene expression to restrict latency and lytic switch, resulting in evasion of immunosurveillance. Furthermore, in BL and EBVaGC, the interaction between the EBV episome and the host genome is evident with respectively unique epigenetic features. While the interaction is associated with suppression of gene expression in BL, the corresponding activity in EBVaGC is linked to activation of gene expression. As EBV establishes a unique latency program in these cancer types, it is possible that EBV utilizes different latency proteins to hijack the epigenetic modulators in the host cells for pathogenesis. Since epigenetic regulation of gene expression is reversible, understanding the precise mechanisms about how EBV dysregulates the epigenetic mechanisms enables us to identify the potential targets for epigenetic therapies. This review summarizes the currently available epigenetic profiles of several well-studied EBV-associated cancers and the relevant distinct mechanisms leading to aberrant epigenetic signatures due to EBV.

## Introduction

Epstein-Barr virus (EBV), also known as human herpesvirus 4, was the first human cancer-associated virus to be discovered (1). A wide range of lymphocytic and epithelial malignancies can be directly associated with EBV infection. Instead of progeny virus production *via* lytic infection, the virus preferentially infects human B-lymphocytes and epigenetically silences the expression of ~80 EBV lytic antigens, presumably by sensing MYC abundance, to maintain a B cell life-long latency (2, 3). This provides the host cell survival advantage to evade the host immunogenic responses. The latency programs established in EBV-associated B-cell or epithelial malignancies, including Burkitt’s lymphoma (BL), Hodgkin’s disease, T cell lymphoma, nasopharyngeal carcinoma (NPC), and ∼10% of cases of gastric cancer (EBVaGC), can be quite different, even within the same type of cancer (4).

The EBV latency proteins include six EBV nuclear antigens (EBNA1, EBNA2, EBNA3A/B/C, EBNA-LP), and three latent membrane proteins (LMP1, LMP2A/B) (5). Studies have demonstrated that some of these latency proteins are strongly associated with oncogenicity. For example, LMP1 has been considered as a classic oncogene for its essential role in primary B cell transformation (6, 7). Similarly, the LMP1 transformation properties, including anchorage-independent growth and invasiveness, could be observed in a SV40T-immortalized nasopharyngeal epithelial (NPE) cell line (NP69SV40T) (8). However, different from EBV-transformed B cells or NPC, the expression of LMP1 in EBVaGC is usually undetectable, suggesting EBV involves different tumorigenic mechanisms in different tissues.

Epigenetics refers to the heritable changes in gene expression independent of changes in the DNA sequence (9, 10). Dysregulation of epigenetic machineries can interrupt the normal expression of tumor suppressor genes (TSGs) and oncogenes, which may ultimately lead to tumorigenesis. Currently, the widely studied epigenetic alterations associated with tumorigenesis include DNA methylation, histone modification, and chromatin conformation alterations. In fact, the impact of EBV in epigenetic regulation is evident and it is involved in promoting development of different cancers. EBNA1, the latent protein involved in maintenance of the EBV episome, is detectable in all types of EBV-associated malignancies and is able to bind to a number of promoter regions of oncogenes and TSGs to regulate their expression in both lymphocytic and epithelial malignant cells (11–13). Another study showed that a subset of breast cancer patients was associated with EBV infection from whom a few critical TSGs were methylated including *BRCA1/2, p14, p16, and hMLH* (14, 15). In contrast, hypermethylation of the tumor-related genes is more common in EBV-negative than in EBV-positive Hodgkin’s lymphoma cases (16). When comparing the EBV-positive and -negative Burkitt lymphomas (BL) cell lines, the contribution of EBV to a specific methylome signature and expression profile associated with BL pathogenesis was reported (17). Although transformation of B lymphocytes by EBV resulted in large-scale hypomethylated blocks (18), the hypermethylation patterns in NPC and EBVaGC could be observed (19, 20), suggesting the distinct epigenetic role of EBV in these cancers.

As the advanced sequencing techniques become more common, the epigenetic profiles including DNA methylation and histone modifications in EBV-associated cancer have been comprehensively investigated nowadays. Hence, this review summarizes and compares the epigenetic profiles of several extensively studied EBV-associated cancers, including NPC, EBVaGC, LCLs, and BL, and their different mechanisms contributing to the unique epigenetic signatures.

## EBV Infection Can Alter DNA Methylation Patterns

DNA methylation usually occurs in CpG dinucleotides by covalently transferring a methyl group (CH_3_) to the cytosine nucleotide, becoming 5-methylcytosine (5mC). This process is catalyzed by a family of DNA methyltransferase (DNMT) enzymes (DNMT1, DNMT3a/c), utilizing S-adenosylmethionine (SAM) as the substrate for the methylation (21). While DNMT3a and DNMT3b, the *de novo* DNMTs, establish a new methylation pattern of unmodified DNA, DNMT1 maintains the DNA methylation pattern during daughter strand DNA synthesis (21). In contrast, the ten-eleven translocation (TET) enzyme can reverse the 5mC to cytosines by oxidizing the 5mC to form 5-hydroxymethylcytosine (5hmC), followed by thymine DNA glycosylase (TDG)-dependent base excision repair (BER) (22). The hypermethylation pattern frequently occurs in the CpG-rich segments, known as a CpG island (CGI). They are abundant in promoter regions, as evidenced by finding roughly 70% of mammalian gene promoters contain CGIs (23). These CGIs are usually unmethylated, allowing transcription initiation. Hypermethylated CGIs of promoters are strongly associated with the gene silencing and dysregulation of the methylation mechanisms is related to various cancer development (24).

### Global DNA Hypermethylation and the Contribution of LMP1 to TSG Silencing in NPC

**Figure 1:**
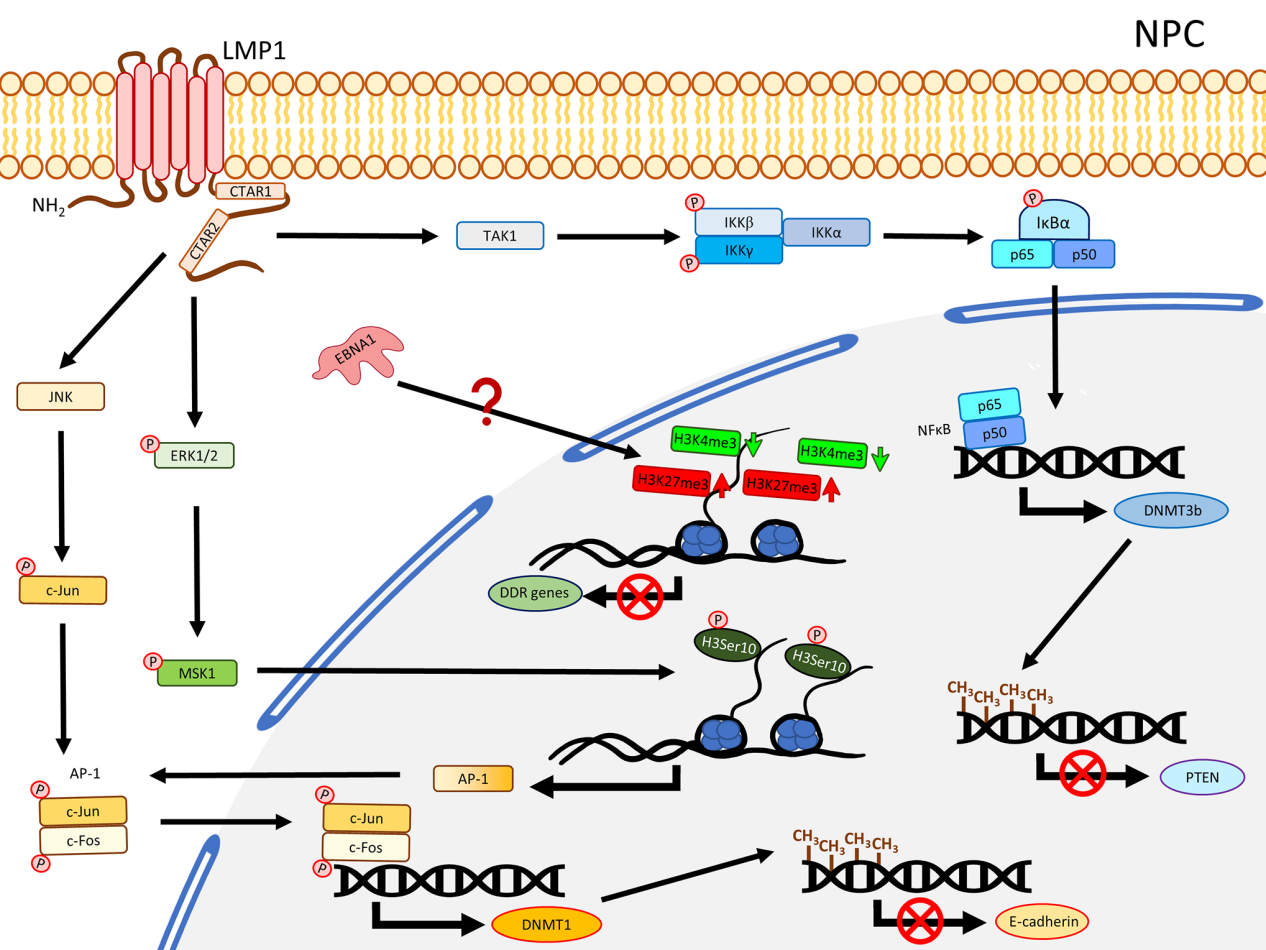
A schematic diagram shows the impact of EBV on epigenetic dysregulation in NPC. LMP1 activates JNK and NFκB signaling pathways to enhance expression of DNMTs, resulting in promoter DNA hypermethylation in the TSGs including *CDH1* (encoding E-cadherin) and *PTEN*. LMP1 may also promote phosphorylation of ERK1/2, resulting in phosphorylation of H3Ser10 to activate the expression of AP-1. Up-regulation of EBNA1 inhibits DDR genes expression probably through dysregulating bivalent switches in NPE cell lines.

Our previous methylome study compared the tumor data from 11 solid NPC primary tumors, which harbored a significant hypermethylation pattern at the CGIs (19). Another methyl-capture sequencing study also identified hypermethylation at the CGIs of some key TSGs (25). In fact, the promoter regions of several critical TSGs have been reported with hypermethylation and validated to be down-regulated in NPC including *RASSF1* (26, 27), *CDKN2A* (28–30), *MIPOL1* (31, 32), *PTPRG* (33, 34), *RRAD* (25, 35), *THY1* (36, 37), *PTEN* (38, 39), *CDH1* (40, 41), and *RARβ2* (42, 43). A study using NPC cell lines demonstrated that the YYD domain at COOH-terminal activation region 2 (CTAR2) of LMP1 is critical to activate the JNK signaling pathway leading to phosphorylation of c-Jun to form an AP-1 complex with c-Jun (homodimer) or c-Fox (heterodimer) to bind to the promoter region of *DNMT1* and enhance the DNMT1 expression (44). The enhanced DNMT1 expression results in hypermethylation of the E-cadherin gene (*CDH1*) and suppresses its expression (44) (**Figure 1**). Likewise, another NPC study also showed that re-expression of LMP1 could enhance the expression of DNMT1 and DNMT3a/b, resulting in methylation of the *CDH1* promoter and reduction of its expression (45). LMP1 was further shown to enhance the expression of DNMT3b through the NFκB pathway, with evidence of p65 binding to the DNMT3b promoter, resulting in hypermethylation of the *PTEN* promoter and, hence, silencing its expression (46) (**Figure 1**). In addition, the significance of LMP1 contributing to DNA methylation was further highlighted by a study in which over-expression of LMP1 in a NPC cell line induced expression of DNMT1 and DNMT3a/b; all these DNMTs are necessary to suppress RARβ2 *via* promoter hypermethylation (42). Although this study also showed that the presence of LMP1 elevated the level of pRB and E2F1, which may be relevant to the enhanced expression of DNMTs, the connection of the Rb-E2F1 pathway to LMP1-mediated DNMTs activation was uncertain in NPC (42). Overall, these studies proposed the importance of LMP1 for the mechanisms regulating the aberrant DNA hypermethylation in NPC to suppress TSGs, as summarized in **Figure 1**.

### Global DNA Hypermethylation and the Contribution of LMP2A to TSG Suppression Through Gene Methylation in EBVaGC

**Figure 2:**
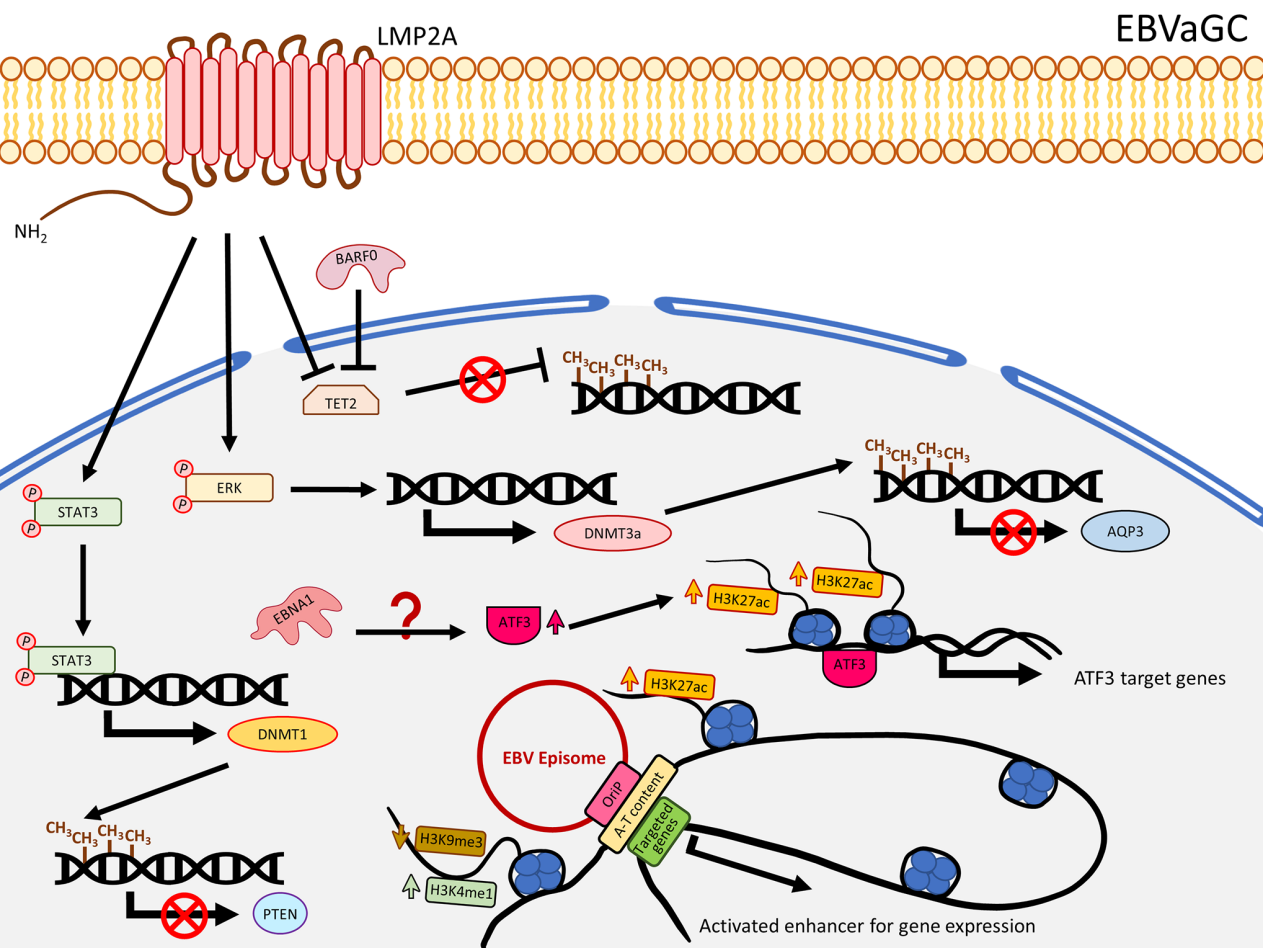
A schematic diagram shows the impact of EBV on epigenetic dysregulation in EBVaGC. LMP2A inhibits TET2 and activates STAT3 and ERK signaling pathways to enhance expression of DNMTs, resulting in promoter DNA hypermethylation to suppress gene expression including *PTEN* and *AQP3*. EBNA1 enhanced the expression of ATF3, which functions as a transcription factor, to promote target gene expression, characterized by gain of H3K27ac. EBV can also interact with the host genome, where A-T content is enriched as well as H3K4me1 and H3K27ac, and reduced H3K9me3 levels to promote gene expression.

In contrast to NPC, LMP1 is usually not expressed in EBVaGC. Nevertheless, this solid tumor also harbors extensive DNA methylation. As reported by The Cancer Genome Atlas (TCGA), EBVaGC is characterized by a higher prevalence of DNA methylation than for other GC cancers. This is commonly known as the EBV-CpG island methylator phenotype (EBV-CIMP) (47). When EBV infects an EBV-negative gastric cancer cell line with low methylation phenotype (MKN7), a DNA hypermethylation pattern similar to that observed in EBVaCG could be obtained (20), suggesting the important role of EBV in the development of the DNA hypermethylation pattern in EBVaGC. Hypermethylation and down-regulation of some TSGs were frequently reported in EBVaGC, including *RASSFI* (48), *CDKN2A* (48–56), *IRF5* (57), *TP73* (56, 58), *CDK2AP2* (51, 52, 55, 56, 59), *WNT5A* (60), *PTEN* (48, 61), and *CDH1* (55, 56, 62). Immunohistochemistry staining also showed the correlation of DNMT1 over-expression. In EBVaGC, EBV contributes to the EBV-CIMP *via* enhancing DNMT1 expression (63). By infecting EBV into an EBV-negative GC cell line, Hino and colleagues showed that LMP2A induced phosphorylation of STAT3 to enhance the expression of DNMT1, leading to silencing of PTEN by promoter hypermethylation in an IL-6 independent manner (61) (**Figure 2**). The role of LMP2A to induce DNMT3a was further confirmed by over-expressing LMP2A in an EBV-negative GC cell line (64). This study demonstrated that LMP2A induces DNMT3a *via* phosphorylation of ERK to hypermethylate the promoter and first exon of *aquaporin 3* (*AQP3*), resulting in down-regulation of AQP3 (64) (**Figure 2**). Since AQP3 was demonstrated to be involved in tumor metastasis and invasion in breast cancer (65), down-regulation of AQP3 in EBVaGC may explain why EBVaGC has a better prognosis compared with other non-EBV-associated GCs (66). Another study infecting EBV in a SV40 immortalized gastric mucosal cell line (GES1) and an EBV-negative GC cell line (MKN7) identified that EBV inhibited the TET family genes, in particular TET2, to protect DNA from demethylation in the host cells (67) (**Figure 2**). A panel of seven human miRNAs was also shown to be up-regulated in the EBV-infected cells (67). When BARF0, LMP2A, or the induced human miRNAs were over-expressed, the expression level of TET2 are significantly suppressed (67), highlighting the involvement of LMP2A in suppression of DNA demethylation. The study also showed that depletion of TET2 without EBV infection was not able to induce *de novo* methylation, whereas knocking down TET2 in EBV-infected cells largely induced *de novo* DNA methylation, indicating TET2 functions as a resistance factor against DNA methylation instead of a contribution factor. Therefore, the *de novo* DNA methylation is likely contributed by EBV (67). To date, the mechanisms about how EBV regulates the DNA methylation in GC are still not fully understood, but the current knowledge summarized here suggests that EBV regulates the DNA methylation mechanisms in the host cell mainly through LMP2A to activate DNMT1/3a and suppress TET2 (68), as summarized in **Figure 2**.

### EBV Transformation of B Cells Is Associated With Hypomethylation of B-Cell Activation Antigens and Hypermethylation of Certain TSGs

**Figure 3:**
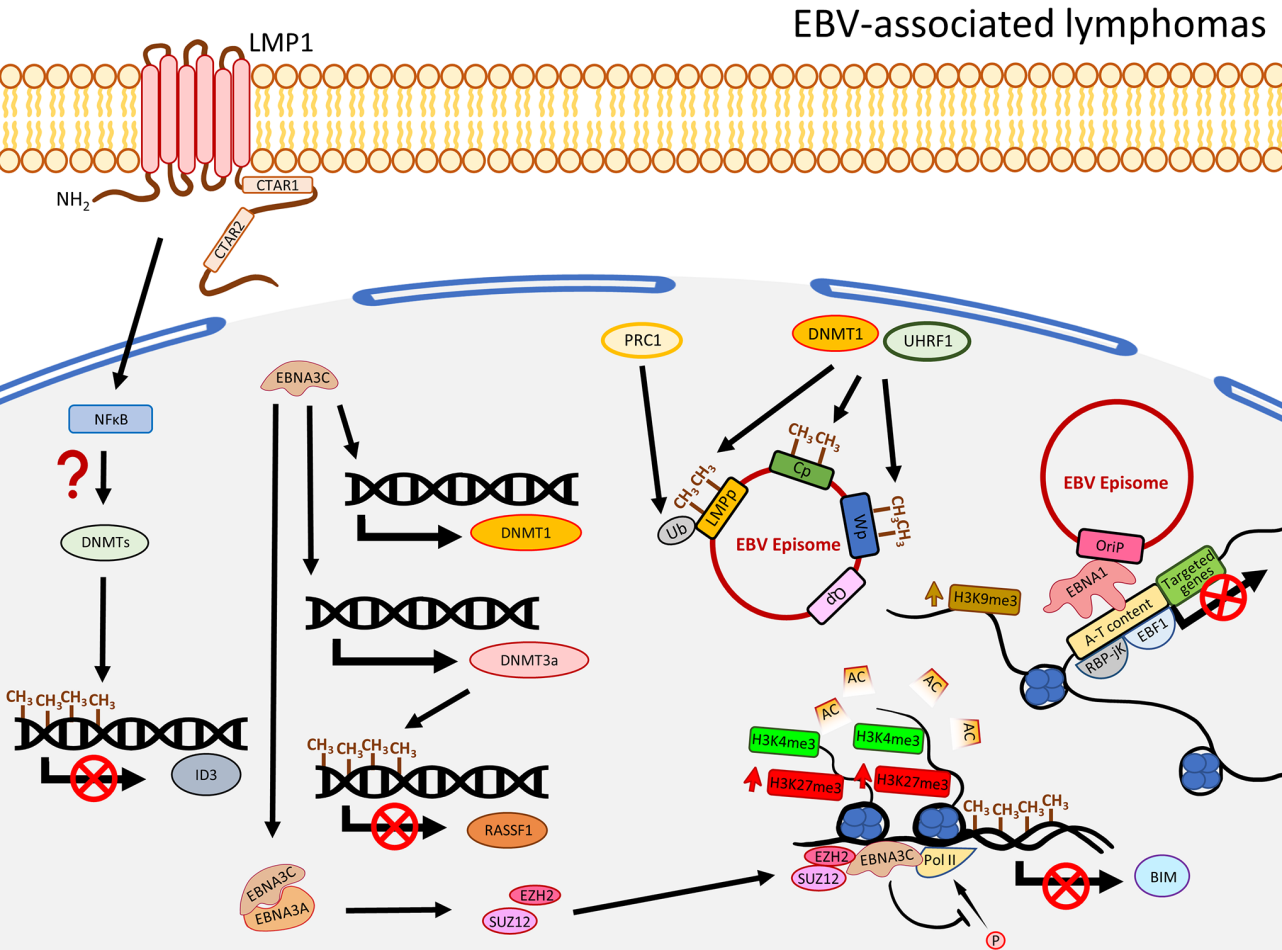
A schematic diagram shows the impact of EBV on epigenetic dysregulation in EBV-associated lymphomas. LMP1 and NFκB are required to suppress the expression of *ID3 via* promoter methylation by a recruitment of the DNMT complex. EBNA3C promotes expression of DNMT3a to induce DNA methylation, resulting in suppression of gene expression including *RASSF1*. EBNA3C also interacts with EBNA3A to regulate the histone bivalent switch to recruit EZH2 and SUZ12 to the *BIM* promoter to regulate the expression, with evidence of the relevant promoter methylation. The EBV C promoter (Cp), W promoter (Wp), and LMP promoter (LMPp) are restricted by the host factors, including DNMT1 and UHRF1, through DNA methylation, or mono-ubiquitination of the LMPp by PRC1 (Ub: H2AK119Ub1). The interactions of EBV and host genome, through EBNA1 tethering, are associated with gene silencing and enriched with enhanced H3K9me3, A-T content, RBP-jK, and EBF1.

EBV infection in B cells is not associated with global DNA hypermethylation. Instead, hypomethylation is commonly observed. EBV-infected primary B cells were shown to undergo demethylation at the promoter regions of a significant number of genes involving B cell activation such as *CD19*, *CD79a*, *BLK*, and *FCER2* (69). Also, the hypomethylation in B cells took place after 24-h post EBV infection, when proliferation started, and many of the hypomethylated domains associated with B cell activation are enriched with Pol II and a NFκB subunit p65 (69), suggesting EBV-associated demethylation induces B cell activation genes through NFκB activity. Although apparent global hypermethylation phenotype in EBV-transformed B cells is not frequently observed (18, 69, 70), notable methylation patterns in some key TSGs are evident (71). By comparing peripheral primary B cells and the corresponding EBV-transformed B cells, Saha and colleagues showed that EBV infection resulted in four methylation patterns for the TSGs, namely gain of methylation, loss of methylation, distinct methylation, and fluctuating methylation (71). Among these groups, 40 TSGs were classified as being in the gain of methylation group and some of them were further validated to be down-regulated in the EBV-infected B cells, including *ATM*, *CDKN1A/B*, *CDKN2A*, *TP53*, *TP73*, and *Rb* (71). Furthermore, at the mRNA level, DNMT3a/b/L and a group of HDACs were shown to be up-regulated in EBV-induced LCLs, suggesting EBV-infected primary B cells may specifically recruit a DNMT3 complex to regulate the TSG expression. Moreover, infection of EBV in B cells resulted in recruitment of a DNMT complex to the promoter of *ID3* to inactivate the expression by hypermethylation. This phenotype was rescued when LMP1 was depleted or when the cells were treated with NFκB inhibitor, suggesting the down-regulation of *ID3* possibly through a LMP1-NFκB-mediated mechanism (17) (**Figure 3**). Similar to LCLs, when comparing to normal germinal center B cells, Burkitt and follicular lymphoma exhibited genome-wide hypomethylation patterns (72). When further comparing EBV-positive and -negative Burkitt lymphoma (BL) cell lines, a distinct distribution of methylated regions was identified. In EBV-positive BL cell lines, while hypomethylation in promoter regions is more common, hypermethylated regions are usually distant from transcriptional start sites (TSS) (17). Another study by over-expressing EBNA3C in an EBV-negative BL cell line, BJAB, demonstrated that EBNA3C induced DNMT1 protein expression and EBNA3C could interact with DNMT1 (73). A recent functional study similarly illustrated that over-expression of EBNA3C could induce DNMT1 and DNMT3a expression, though the effect of DNMT1 was not obvious (74). The enhanced expression of DNMT3a could also be observed in LCLs, when compared with BJAB. This study further showed that the expression of RASSF1 was down-regulated by promoter hypermethylation in LCLs and EBNA3C-over-expressing BJAB (74), suggested EBNA3C down-regulated RASSF1 through enhancing DNMT activity to methylate the *RASSF1* promoter. The EBV-mediated up-regulation of DNMT3a was further supported by an observation of an up-regulation of DNMT3a in EBV-infected germinal center-derived B cells and HL cell lines, even though DNMT1 and DNMT3b were shown to be down-regulated (75). The above studies emphasize that LMP1 and EBNAs are essential in the DNMT-mediated host gene expression regulation. The effects on DNMTs expression in different EBV-associated lymphomas are varied with the only consistency being that EBV infection in peripheral primary B cells, germinal center-derived B cells, LCLs, and EBNA3C-over-expressing BL cell lines indistinguishably showed an increased expression of DNMT3a (**Figure 3**).

In addition to regulating the host gene expression, EBV could also usurp the host factors to modulate its own gene expression. A recent study utilized a genome-scale CRISPR-Cas9 screen to identify that UHRF1 and DNMT1 are critical to restrict EBNAs and LMPs expression through DNA methylation in the C promoter (Cp) and LMP promoters (LMPp) to maintain latency I in BL cells (76). The same study also showed the importance of the subunits of PRC1 to suppress LMPp and Cp by histone 2A lysine 119 mono-ubiquitination (H2AK119Ub), and further demonstrated the viral DNA methylation was initiated by DNMT3B and maintained by DNMT1 (76). On the other hand, the significant role of MYC, as a lytic repressor by inhibiting oriLyt association with the BZLF1 promoter, was demonstrated. Taken together, it seems that EBV not only regulates the host gene expression, it also interplays with the host factors to epigenetically regulate its own viral gene expression in order to restrict the latent and lytic switch. Limiting the expression of the latent and lytic oncoproteins makes it possible for the infected B cells to evade immune surveillance and contribute to EBV pathogenesis and oncogenesis.

## EBV Infection Results in Aberrant Histone Modifications

Aberrant histone modification is another epigenetic feature associated with cancer development (77, 78). The N-terminal tails of histones are subject to a variety of post-translational modifications (PTMs) including methylation, acetylation, ubiquitylation, sumoylation, and phosphorylation on specific residues (79, 80). The side chains of lysine on histone H3 is a site where various modifications frequently occur (81). The well-documented histone modifications include, but are not limited to, acetylation of H3 at lysine 27 (H3K27ac), trimethylation of H3 at lysine 4 (H3K4me3), at lysine 27 (H3K27me3), and at lysine 9 (H3K9me3). Lysine acetyltransferases (KATs), also known as histone acetyltransferases (HATs), are the enzymes that recruit acetyl groups to histone lysine residues. Introducing a acetyl group to the residue neutralizes the positive charge on the lysine and weakens the electrostatic interaction between negatively charged DNA and histones, resulting in loosening of chromatin structure to be accessible to transcription factors (TFs) (78, 79). Hence, H3K27ac is usually localized to the enhancer and promoter regions and is associated with active transcription (78, 82). Histone deacetylases (HDACs), in contrast, are responsible for removal of the acetyl group (78). The balance activities between HATs and HDACs maintain the dynamic acetylation equilibrium and, hence, regulate the gene expression (83, 84). Unlike acetylation, methylation of a histone does not alter the charge. Depending on the site and degree of methylation, methylation of a histone can result in either activation or repression of gene expression. For instance, the H3K4me1 and H3K4me3 are transcriptional activation marks, while H3K9me3 and H3K27me3 are typically found in the proximity of transcription suppressive sites (85). Similar to the DNA methylation, SAM was used as the methyl group donor by histone methyltransferases (HMTs), and is removed by histone demethylases (HDMs) (86). Among the HMTs, the polycomb repressive complex 2 (PRC2) is widely known as an H3K27 methyltransferase, which is composed of four core subunits including EZH1/2, SUZ12, EED, and RbAp46/48 (87, 88). Notably, H3K4me3 and H3K27me3 are reported to coexist in the promoters as a bivalent mark to fine-tune gene expression (89–91). The bivalent domains were first described in embryonic stem cells (ESCs) by Bradley and colleagues to regulate developmental gene expression (90). They observed that expression of some developmental genes was low or not expressed at all with the presence of the bivalent marks. However, the expression of the corresponding genes was elevated upon differentiation and exhibited reduction of H3K27me3 in the bivalent domains. It was suggested that the bivalent domains silenced developmental genes in ESCs, while keeping them poised for activation by loss of H3K27me3 (90). In addition to regulating the developmental gene expression, deregulation of the bivalent marks is also associated with a variety of cancers (91–94). Phosphorylation of histone H3 at Ser 10 (H3Ser10), a marker of mitosis, is critical for chromosome condensation, which was previously shown to be regulated by mitogen- and stress-activated kinase 1 (MSK1) (95–97). Phosphorylation of H3Ser10 (pH3Ser10) was also demonstrated to be crucial for cell transformation (96, 98), implicating the association of pH3Ser10 in cancer development.

### Elevation of H3K27me3 and the Modulation of Gene Expression *via* Histone Modification in NPC

By comparing NPC tissues and the adjacent non-tumor sites in the nasopharynx, a study showed that the level of H3K27me3 was significantly higher in NPC and positively correlated with tumor metastasis, advanced clinical stage, chemo-radioresistance, and survival time (99). The level of H3K27me3 was, hence, proposed as a biomarker for NPC patient survival and chemo-radioresistance (99). The elevated level of H3K27me3 is possibly attributable to the enhanced activity of a PRC2 subunit, EZH2, in NPC (100). A ChIP-Seq study using NPC tumor tissues and xenografts targeting H3K27ac identified a distinct group of super enhancers (SE), among which an oncogene, ETV6, was further demonstrated to be up-regulated and correlated with poor survival in NPC patients (101). Also, the enhancer H3K27ac signals were enriched in NFκB-p65 motif (101). Since it is well-reported that the NFκB pathway is constitutively activated by LMP1 in NPC, it is possible that LMP1 regulates the H3K27ac enhancer near NFκB-p65 promoter to activate the NFκB pathway. Another study revealed that there was a positive correlation between LMP1 and pH3Ser10. A significantly higher level of pH3Ser10 in the poorly differentiated NPC tissues compared with chronic nasopharygitis tissues and normal nasopharynx tissues was observed (102). The same study further showed that over-expression of LMP1 in an NPC cell line increased the level of pERK1/2, pMSK1, pH3Ser10, and is associated with increased AP-1 promoter activity. Suppression of ERK1/2 and MSK1 by a small inhibitor and shRNA reduced the level of pH3Ser10 and AP-1 promoter activity (102), suggesting LMP1 regulates pH3Ser10 though the ERK/MSK1 pathway to promote AP-1 expression (**Figure 1**). As AP-1 was also demonstrated to regulate DNMT1 expression for methylation of the E-cadherin promoter in the presence of LMP1, it is possible a cross-talk exists in which LMP1 enhances the expression of AP-1 through phosphorylation of H3Ser10 at the promoter of AP-1, providing sufficient AP-1 for JNK/AP-1 pathway to up-regulate DNMT1 (**Figure 1**). In addition, our previous ChIP-Seq study comparing two pairs of EBV-positive and -negative NPE cell lines illustrated that EBV infection dysregulated histone bivalent switches, showing reduction of H3K4me3 and gain of H3K27me3, to suppress DNA damage repair (DDR) gene expression in a methylation-independent manner (103). The same study further showed that over-expression of EBNA1 could inhibit the expression of DDR genes (103). It seems that EBV could dysregulate the histone modifications in the precancerous host cells to suppress the key cancer-related genes without the involvement of DNA methylation. Nevertheless, more studies are needed to ascertain the direct contribution of EBNA1 to regulate the bivalent switches and, hence, suppress DNA damage repair genes in NPC. Furthermore, the *de novo* methylated loci identified from NPC remarkably overlap with the bivalent marks derived from human ESCs (19), implicating the possible correlation of bivalent marks and DNA methylation in NPC development.

### Redistribution of Histone Marks and Interaction of the EBV Episome With the Host Cell Genome Is Associated With Activation of Host Cell Gene Expression in EBVaGC

A ChIP-Seq study analyzing H3K4me1, H3K4me3, H3K27ac, H3K9me3, and H3K27me3 showed that EBV infection in GC cell lines redistributed these histone marks in the promoter and enhancer regions (68). While the repressive mark H3K27me3 was shown to be higher in NPC (99), Atsushi *et al*. demonstrated that EBV infection in GC cell lines significantly reduced H3K27me3 level around the TSS (68), consistent with another EBVaGC ChIP-Seq study showing EBV infection rarely increased H3K27me3 signal in the promoter regions (104). Furthermore, they identified EBV infection significantly contributed to gain of H3K27ac in the enhancer regions and those simultaneously associated with reduction of H3K9me3 or unchanged level of H3K27me3 are positively correlated with gene expression (68). The enhancer activated genes were significantly enriched with some cancer-related pathways, involving in cell proliferation and differentiation (68). The elevated H3K27ac signal may be due to the up-regulation of EP300 and down-regulation of HDACs in the EBV-infected GC cell lines, as shown in an RNA-Seq study (68), suggesting EBV dysregulates the balance of HAT (EP300) and HDACs, and consequently contributing to up-regulation of H3K27ac signals for tumorigenesis (**Figure 2**). The importance of EBV-regulated H3K27ac in GC cells was further emphasized by a recent study showing that EBV infection in MKN7 increased the expression of ATF3, and a ChIP-Seq analysis further showed that the binding sites of ATF3 are associated with enhanced H3K27ac signals (105). The neighboring genes of ATF3 binding sites were suggested to be ATF3 target genes. When ATF3 was knocked down in EBVaGC cell lines (SNU719 and NCC24), these ATF3 target genes were down-regulated, resulting in growth suppression and apoptosis (105). The upregulation of ATF3 may be contributed by EBNA1, as evident by an EBNA1 over-expression analysis in MKN7 (105) (**Figure 2**). Another ChIP-Seq study identified the correlation of promoter DNA methylation and histone modifications in EBV-infected GC cell lines (104). Sayaka *et al*. analyzed DNA methylation and alteration of H3K4me3, H3K27ac, and H3K27me3, revealing that although a number of genes were methylated after EBV infection, the expression of these DNA methylation-induced genes was not completely silenced, depending on if they were classified as DNA methylation-sensitive genes or DNA methylation-resistant genes (104). While the whole promoter in DNA methylation-sensitive genes is methylated to suppress gene expression, DNA methylation-resistant genes acquire DNA methylation merely around the TSS and retain gene expression after EBV infection. The gene ontology analysis showed that the DNA methylation-sensitive genes are enriched with regulation of cell differentiation, migration, proliferation, and cell adhesion, but DNA repair and DNA metabolic processes are enriched for DNA methylation-resistant genes (104). From the same study, they also found that the TSS in DNA methylation-resistant genes was protected from methylation by active histone marks (H3K4me3 and H3K27ac) allowing them to be expressed, including the DDR genes (*MLH1, MSH2*, and *MSH6*), but loss of these marks makes them susceptible to DNA methylation (104). It is noteworthy that DNA methylation-sensitive genes, including *CDKN2A*, *CDH1*, and *RHOB*, are associated with loss of H3K27me3 or reduction of H3K4me3 and H3K27ac (104). On the whole, it is plausible that EBV-associated DNA methylation could be modulated by redistribution of these histone marks to regulate the gene expression.

The EBV viral genome predominantly persists in the nucleus in a circular episomal state instead of integration into the host genome, suggesting that EBV episome may interact with the host genome to, in a certain extent, influence gene expression. Interaction of EBV episome and the host genome, in fact, could result in redistribution of histone marks. Using a panel of 14 GC cell lines, including three EBV positive, two immortalized gastric epithelial cell lines, and EBV-positive primary GC tissues, a recent chromosome conformation capture combined with high-throughput sequencing (Hi-C) study showed that the EBV genome, in particular the oriP region, preferentially associated with human chromosomes 2, 3, 4, 6, 7, and 13, and these EBV-interacting regions usually exhibited higher A-T content, reduced H3K9me3, as well as higher H3K27ac or H3K4me1 signals (106) (**Figure 2**). This study further showed that the domain and loop structure alterations by EBV infection could induce cancer-related gene expression including *KLF5, TGFBR2*, and *MZT1* (106), suggesting the role of EBV in the regulation of gene expression through histone modification and chromatin conformation alteration.

### Global Reduction of H3K9//27me3 and H4K20me3 in EBV-Transformed LCLs Is Associated With Host Cell Gene Suppression in BL

The global level of H3K27me3 in LCLs is different from NPC, but similar to EBVaGC, showing significant reduction (107). The global reduction of H3K27me3 may be due to the LMP1-mediated up-regulation of a histone demethylase, Jumonji Domain-Containing Protein 3 (JMJD3), as shown in EBV-associated Hodgkin’s lymphoma (108). However, the apparent up-regulation of JMJD3 was not observable in another study infecting EBV in EBV-negative BL cell lines (109). Meanwhile, global losses of H3K9me3 and H4K20me3 were also observed in LCLs (107). The reduction of H3K27me3 and H3K9me3 was further shown to be associated with down-regulation of a cluster of *HOX* and *ZNF* genes, respectively (107). However, similar to NPC, a great number of SEs were identified to regulate gene expression in LCLs (110). By analyzing ChIP-Seq data, Hufeng and colleagues showed that the enhancer regions of some oncogenes harbor significantly higher signals of NFκB subunits (RelA/B, cRel, p52, and p50), TFs, H3K27ac, and EBNAs (EBNA2, EBNALP, and EBNA3A/C) and these regions were, therefore, classified as EBV super-enhancers (EBV-SEs) (110). Some oncogenes associated with EBV-SEs, including MYC and BCL2, were further shown to be excessively upregulated (110). In fact, poor clinical outcomes for lymphomas patients with co-expression of MYC and BCL2 were previously reported (111). The epigenetic regulation of gene expression by EBV was further supported by Kostas *et al*., showing that the expression of a pro-apoptotic protein, BIM, was significantly inhibited in EBV-infected B cells and was associated with reduced promoter acetylation on histones H3 (K9, K14) and H4 (K5, K8, K12, and K16), increased H3K27me, and DNA methylation (112). Subsequently, EBNA3A/C was demonstrated to recruit EZH2 and SUZ12 (PRC2 subunits) to regulate histone bivalent switches in the promoter region of BIM, showing gain of H3K27me3 without changing H3K4me3, leading to down-regulation of BIM (109). This study has further shown that EZH2, SUZ12, and EBNA3C were enriched at the *BIM* promoter region (109). The down-regulation of *BIM* may be due to the inhibition of phosphorylation of Pol II by the presence of EBNA3A/C (109). Also, EBNA3A and EBNA3C can be co-immunoprecipitated, suggesting these EBNAs may act directly on the site with the PRC2 complex for the aberrant histone modifications at *BIM* promoters (109) (**Figure 3**). As MYC could trigger apoptosis by inducing BIM, inactivating BIM by EBV could alleviate the BIM-mediated apoptotic stress and, hence, promote carcinogenesis (113, 114). The importance of EBNA3A/C in suppression of another TSG, p16 (*CDKN2A*), was highlighted by Lenka and colleagues as well (115). They observed that the expression of p16 was inhibited in LCLs with the presence of high H3K27me3 and low H3K4me3 at the corresponding promoter regions, suggesting the involvement of histone bivalent marks. Inactivation of EBNA3A or EBNA3C de-repressed p16 and was associated with reduction of H3K27me3 and gain of H3K4me3 at the exon 1 region (115). As DNA methylation of *CDKN2A* promoter was previously reported in LCLs (71), the methylation may be due to the presence of the H3K27me3 contributed by the PCR2 complex in which the subunit EZH2 was known to enable recruitment of DNMTs (116). Although C-terminal binding protein (CtBP) was shown to interact with EBNA3A/C in primary rat embryo fibroblasts and the necessity of CtBP to inhibit p16 in LCLs was proven, the interaction of CtBP with EBNA3A/C to inhibit p16 in LCLs needs further investigation (115).

The EBV genome was further shown to interact with the host genome enriched with EBNA1, EBF1, and RBP-jK binding sites (117). Another study found that EBV interacts with repressive compartments of the host genome during latency, but with active compartments during lytic reactivation (118) in BL cell lines. Strikingly, although EBV and host genome interaction sites are similar to those demonstrated in EBVaGC exhibiting A-T rich content, this interaction in BL is usually enriched with H3K9me3, and, hence, inhibits gene expression (106, 117). This implies that the EBV episome interacting with the host genome in epithelial and B cells results in different influence on gene expression. While the interaction in GC induces gene expression through reduction of H3K9me3 and is associated with active marks (H3K27ac or H3K4me1), the interaction in BL *via* EBNA1 binding results in repressive of gene expression and is associated with H3K9me3 (106, 117). **Figure 3** summarizes the EBNA-associated mechanisms to regulate the host histone status and gene expression.

## Conclusions

To summarize, it is evident that EBV infection results in hypermethylation in NPC and EBVaGC, driven by LMP1 and LMP2A, respectively, and is associated with down-regulation of TSGs. Although promoter methylation of a few TGSs in EBV-infected B cells is observable, it is unlikely that EBV contributes to global hypermethylation in B cells. With regard to the histone modification status, while H3K27me3 is globally prevalent in NPC, a critical elevation level of H3K27me3 is not obvious in EBVaGC and EBV-infected B cells. The distinct methylation and histone modification status in the relevant cancer types is summarized in **Table 1**. Also, histone bivalent switches in NPC and LCLs are critical to regulate gene expression and it is probably contributed by the EBNAs activities. It is also evident that EBV utilizes the host factors to restrict the viral gene expression to evade immune surveillance. Moreover, while interaction of EBV and the host genome is associated with elevation of activation marks to allow gene expression in EBVaGC, such interaction in BL cells is evident with enhanced H3K9me3 to suppress gene expression. The currently available EBV epigenetic studies convincingly suggested that EBV utilizes different mechanisms to hijack the host epigenetic system in different tissue types, highlighting the critical role of EBV latent proteins LMP1, LMP2A, and EBNA3C in NPC, EBVaGC, and EBV-associated lymphomas, respectively. Therefore, precisely targeting of different LMPs and EBNAs in different malignant tissues may restore these epigenetic abnormalities and, hence, improve the epigenetic therapies.

**Table 1:** Methylation and histone modification features for NPC, EBVaGC, LCLs, and BL.

Cancer types	Latency program	Distinct methylation status	Distinct histone modifications	Ref.
NPC	II	Global hypermethylation including critical TSGs	Globally enhanced H3K27me3	(19, 25–43, 99)
EBVaGC	I (>50% express LMP2A)	Global hypermethylation including critical TSGs	Reduction of H3K27me3 around TSS; EBV genome interacts with host genome in which H3K9me3 is less frequent, while H3K27ac or H3K4me1 is enriched to activate gene expression	(20, 47–62, 68, 106)
LCLs	III	Global hypomethylation including B cell activation genes, and hypermethylation of TSGs	Global reduction of H3K27me3, H3K9me3, and H4K20me3	(18, 69–71, 107)
BL	I	Global hypomethylation; hypomethylation near promoters while hypermethylation is distant from TSS	EBV genome interacts with host genome in which H3K9me3 is enriched to suppress gene expression	(17, 72, 106, 117, 118)

## Author Contributions

MML: conceptualized and wrote the manuscript. MLL: guidelines and proofreading. All authors contributed to the article and approved the submitted version.

## Conflict of Interest

The authors declare that the research was conducted in the absence of any commercial or financial relationships that could be construed as a potential conflict of interest.
